# Short birth interval and associated factors among women who gave birth in the last three years in Dembecha district, Northwest Ethiopia

**DOI:** 10.1371/journal.pone.0272612

**Published:** 2022-08-23

**Authors:** Mastewal Belayneh Aklil, Kiber Temesgen Anteneh, Tibeb Zena Debele, Wubedle Zelalem Temesgan

**Affiliations:** Department of Clinical Midwifery, School of midwifery, College of Medicine and Health Sciences, University of Gondar, Gondar, Ethiopia; Hosanna College of Health Sciences, ETHIOPIA

## Abstract

**Background:**

Maternal and child mortality remains a major public health problem in Ethiopia. Improving short birth interval practice is a main strategy to reduce neonatal mortality, maternal mortality, adverse pregnancy outcomes, high fertility rate, and enhance economic development efforts. However, there has been limited study done regarding short birth intervals in the study area. Therefore, this study aimed to assess short birth intervals and associated factors among women who gave birth in the last three years in Dembecha district, Northwest Ethiopia, 2019.

**Method:**

A community-based cross-sectional study was conducted among 880 reproductive-age multipara mothers using a stratified cluster sampling technique. The data were collected by face-to-face interviews through pretested and semi-structured questionnaires. Bivariable and multivariable logistic regression model was fitted. Variables with a p-value ≤0.05 were considered statistically significant factors. Adjusted odds ratio with a 95% confidence interval was used to report the association between predictors and the outcome variable.

**Results:**

The prevalence of short birth interval was found to be 43.4% (95% CI: 40.2, 46.9). Husband education (able to read and write) [AOR:2.81,(95% CI:1.04,7.85)], wealth index (lowest quartile) [AOR:3.75,(95% CI:2.35,5.97), residence (urban) [AOR:3.20,(95% CI:1.62,6.33)],age at first marriage (15–17 years old) [AOR: 1.65,(95% CI:1.15, 2.26),and non-use of contraceptive [AOR: 8.78, (95% CI: 6.18, 12.47) were statistically significant variables.

**Conclusion:**

The study revealed that the prevalence of short birth intervals among multipara women is found to be high. Husband education, wealth index, urban residence, age at first marriage, and non-use of contraceptives were variables significantly associated with short birth intervals. Hence, to overcome the problem the focus should be on increasing family planning utilization, avoiding early marriage, strengthening paternal education, and improving family income.

## Introduction

Birth interval is the length of time between two successive live births [1]. According to the World health organization (WHO), a short birth interval is defined as an interval between two consecutive live births of less than 33 months [2]. Evidences suggested that successive birth intervals should be three to five years extended to ensure the best protection for mothers’ and newborns’ health [3–6]. As different studies showed, a relationship exists between shorter birth intervals and high infant, child, and maternal mortality and morbidity [7–9].

Worldwide, around 25% of births occur at intervals of less than 24 months. About 33% of short birth intervals occur in Central Asian States and it is 57% in Sub-Saharan Africa [10]. In Ethiopia, 53% of women had a short birth interval [11].

Studies suggested that women who have experienced a short birth interval were prone to several health problems. The problems inflict harm on both mothers and their children. Some of the health problems faced by the mother include: antepartum hemorrhage, anemia, premature rupture of membrane, postpartum hemorrhage, failure of vaginal birth after caesarian delivery, uterine rupture, and maternal death [5,6,12–14]. These complications occur as a result of nutritional depletion, cervical inefficiency, vertical transmission of infection, and incomplete healing of the uterine scar from previous caesarian delivery [7].

Short birth interval (i.e. <24 months) increases the infant mortality by 60% as compared to birth intervals of 4–5 years. This is due to an increment of foliate depletion, low birth weight, malnourishment, small for gestational age, suboptimal breastfeeding due to pregnancy overlap, sibling competition, and preterm delivery [4,7,8,15]. A meta-analysis conducted in Ethiopia also supported this finding. However, if a birth interval can be extended at least for two years, infant mortality would be reduced by 50% [8,16].

In addition to health-related complications, a short birth interval is one of the contributing factors for an increment of the total fertility rate and challenges the developmental efforts of a country [17].Though total fertility rate (TFR) has decreased from 4.8 in 2011 to 4.6 births per woman in 2016, it still requires much effort to bring significant change in the population growth [8,20,21].

In Ethiopia, there are limited studies done on short birth intervals, particularly in the study area. Therefore, understanding birth interval and its determinants is critical for a country like Ethiopia to design and improve child spacing programs which in turn will improve maternal and child health. Hence, this study was aimed to reveal the prevalence and factors associated with short birth intervals in Dembecha district.

## Methods

### Study design and setting

A community-based cross-sectional study was conducted from September 20 to October 20, 2019 in Dembecha district. Dembecha district is located in the West Gojjam zone of Amhara Regional State, Northwest Ethiopia,350 km away from Addis Ababa, the capital city of Ethiopia, and 215 km away from Bahir Dar, the capital city of Amhara Regional State. The town has 4 urban and 31 rural kebeles, which are the smallest administrative units of the country. According to the population projection of Ethiopia from 2014–2017, for all regions, the total population of Dembecha district was estimated to be 151,023. Among these, 75,464 were females [18].

### Sample size and sampling technique

The sample size was determined by a single population proportion formula by taking 51.2% proportion of short birth intervals from a previous study conducted in the Oromia region [6], a 95% confidence interval, 5% margin of error and design effect 2. The study subjects were selected by using stratified cluster sampling technique after estimation of targeted groups was done in the selected kebeles. Accordingly, the kebeles were stratified as rural and urban kebeles. Then proportional to size allocation techniques were used in determining the number of kebeles included in each stratum. Eight rural and one urban kebeles were selected using simple random sampling from 31 rural and 4 urban kebeles respectively. All eligible participants in the selected cluster were included in the study, making the final sample size 895. For 10 participants have failed to complete the questionnaire and 5 participants have refused to participate in the study, the response rate of the study was 98.3% and the final sample size was 880. A sample of 880 women of any of all marital statuses, between the ages of 15 and 49 years, with two or more children and whose last child was born within the last three years before the survey were included in the study, and women who have a history of abortion between the last child and index child, were excluded from this study.

### Operational definitions

#### Short birth interval

Refers to an interval less than 33 months of a birth interval between the birth of the last child and the immediately preceding live birth to the mother [2,19].

#### Index child

It is defined as the child born immediately prior to a subsequent child [5].

#### Knowledgeable

Those respondents who scored at least 60% of the knowledge questions about a short birth interval correctly were considered knowledgeable [20].

#### Negative attitudes

Those respondents who scored above the mean in answering the attitude questions were taken as having a negative attitude towards a short birth interval [1].

### Data collection instrument and process

Data were collected by face-to-face interviews using a semi-structured and pre-tested questionnaire which was first prepared in English and translated to Amharic and then translated back to English. Nine BSc midwifery degree holders were involved in the data collection while two MSc holders supervised the data collection process in each cluster. Before data collection, written informed consent was obtained from study participants who were over the age of 18 years. Whereas, for those participants who were under the age of 18 years, written informed consent was obtained from their parents/guardians and verbal assent was obtained from study participants for participation. Throughout the course of the data collection, regular meetings were held among the data collectors, supervisors, and the principal investigator. The collected data were reviewed and checked for completeness before data entry and the incomplete data were discarded.

### Data analysis

The outcome variable for this study was a short birth interval. In this study, only the last birth interval was measured and the last birth was within the last three years to reduce recall bias. This was measured by assessing the length of time between the preceding birth and the current (last) birth. The participants’ answers were categorized according to the WHO-recommended birth interval (33 months) and coded 1 if the birth interval was <33 months and 0 if the birth interval was ≥33 months.

The knowledge level of the respondents was assessed using 8 items. The respondents’ level of knowledge about birth interval was reported as good knowledge if the study participants correctly responded to more than or equal to 60% of knowledge assessment tools. The attitude of respondents was assessed using 17 items and categorized as positive if they responded below the mean of attitude-related items.

Data were entered into Epi Info version 7.1.5.2 and exported to Statistical Package for Social Science (SPSS) version 20 for analysis. Bivariable and multivariable logistic regression was used to determine factors associated with short birth intervals. Variables with a P-value of less than 0.2 in the bivariable analysis were included in the multivariable logistic regression to adjust for possible confounders. A p-value ≤ 0.05 with a 95% confidence interval for the adjusted odds ratio was used to determine the level of significance.

### Ethics approval and consent to participate

Ethical clearance was obtained from the School of Midwifery under the delegation of the Ethical Review Board of the University of Gondar. A formal letter of cooperation was written to each selected kebeles by Dembecha district health office. Written informed consent was obtained from study participants who were over the age of 18, and assent was obtained from those participants who were under the age of 18, after they had been informed about the objective of the study. In the consent, statements about the potential risk, benefit, and confidentiality were included. Accordingly, the Ethics committee approval was obtained for this written consent.

## Results

### Socio-demographic characteristics

A total of 895 mothers were eligible in the selected kebeles; from which, 880 participants responded or participated in the study, making a response rate of 98.3%. The median age of the respondents was 34 years with an IQR of 8 years. Half (50.1%) of the total participants had no formal education **(**Table 1**)**.

**Table 1:** Socio-demographic characteristic of study participants in Dembecha district, Northwest Ethiopia, 2019 (n = 880).

Variables	Frequency	Percent (%)
**Age of mother**		
15–24	36	4.0
25–29	202	23.0
30–34	239	27.2
35–39	263	29.9
40–49	140	15.9
**Marital status**		
Married	849	96.5
Single	19	2.2
Ever married**	12	1.3
**Religion**		
Orthodox	841	95.6
Muslim	31	3.5
Protestant	8	0.9
**Maternal education**		
Unable to read and write	441	50.1
Able to read and write	281	31.9
Primary education	96	10.9
Secondary education	49	5.6
College education and above	13	1.5
**Maternal occupation**		
Employed	41	4.7
Housewife	163	18.5
Merchant	32	3.6
Student	7	0.8
Farmer	624	70.9
Daily laborer	13	1.5
**Residence**		
Rural	804	91.4
Urban	76	8.6
**Husband education** (n = 849)		
Unable to read and write	145	17.1
Able to read and write	523	61.6
Primary education	101	11.9
Secondary education	53	6.2
College education and above	27	3.2
**Husband occupation** (n = 849)		
Employed	61	7.2
Merchant	47	5.6
Farmer	724	85.2
Daily laborer	17	2.0
**Wealth index quartile**		
Lowest	172	20.0
Second	180	20.1
Third	177	20.0
Highest	351	39.9

Ever married** refers to divorced and widowed.

### Reproductive characteristics of study participants

More than half (53.4%) of mothers were first married at the age of 15–17 years. A parity of four was seen in 525 (59.7%) of the participants. Four hundred eleven (46.7%) study participants have 3 or 4 alive children. More than half 465 (52.8%) of respondents delivered their index child in the age range of 20–29 years. Over 3/4^th^ (87%) of respondents had antenatal (ANC) follow-ups for the index child. Most of the respondents 590 (67%) delivered their index child at the health institutions. About 568 (66.9%) of the respondents breastfed their child for 24 months and above **(**Table 2**)**.

**Table 2:** Reproductive history of study participants in Dembecha districts, North West Ethiopia, 2019.

Variables	Frequency	Percent (%)
**Age at first marriage** (n = 859)		
15–17	470	53.4
≥18	389	44.2
**Number of parity** (n = 880)		
2	132	15.0
3	223	25.3
≥4	525	59.7
**No of alive children currently** (n = 880)		
1–2	134	15.2
3–4	411	46.7
≥5	335	38.1
**Age at the delivery of index child** (n = 880)		
15–19	21	2.4
20–29	465	52.8
30–39	358	40.7
40–49	36	4.1
**ANC visit at index child** (n = 880)		
Yes	765	86.9
No	115	13.1
**No of ANC visit** (n = 765)		
1–4	732	95.7
≥5	33	4.3
Gained BI* information during ANC (n = 765)		
Yes	721	94.2
No	44	5.8
**Place of delivery at index child** (n = 880)		
Health institution	590	67.0
Home	290	33.0
**Sex of index child** (n = 880)		
Female	517	58.8
Male	363	41.2
**Number of deliveries at index child** (n = 880)		
Single	856	97.3
Multiple	24	2.7
**Survival status of index child** (n = 880)		
Yes	854	97.0
No	26	3.0
**Breastfed index child** (n = 880)		
Yes	849	96.5
No	31	3.5
**Duration of breastfeeding** (n = 849)		
0–12 months	107	12.6
13-23months	174	20.5
≥24months	568	66.9
**Planned last child** (n = 880)		
Yes	855	97.2
No	25	2.8
**Want another child after index child (n = 880)**		
Yes	757	86.0
No	123	14.0
**Preferred birth interval after index child** (n = 757)		
<24 months	29	3.8
24-35moths	362	47.8
≥36months	366	48.4
**Who decides birth intervals (n = 880)**		
Herself	9	1.0
Husband	174	19.8
Herself and husband	697	79.2
**Husband idea for spacing** (n = 849)		
Supports	750	88.2
Opposes	92	10.9
No idea	7	0.9
**Length of birth interval** (n = 880)		
Short birth interval	382	43.4
Not short birth interval	498	56.6
**Contraceptive use after index child** (n = 880)		
Yes	574	65.2
No	306	34.8
**Aim of contraceptive use** (n = 574)		
Spacing	434	75.6
Limiting	140	24.4
**Communicate with husband** (n = 880)		
Yes	670	76.1
No	210	23.9

BI*-Birth interval.

### Knowledge and attitude of the participants

More than three fourths (87.2%) of the respondents had information about short birth intervals, and the main source of information for the majority (66.3%) of the respondents was health care providers. Among the respondents who have information on birth intervals, 67.3% of them mentioned that a short birth interval was considered when it was less than 33 months **(**Table 3). From the total participants, 587(66.8%) were knowledgeable, whereas, 541(61.5%) respondents had negative attitudes towards short birth intervals **(**Table 4).

**Table 3:** Knowledge of study participants about short birth interval in Dembecha, northwest Ethiopia, 2019.

Variables	Frequency	Percent (%)
**Have you ever heard about short birth interval?**		
No	113	12.8
Yes	767	87.2
**How many months do you think is short birth interval? (n = 767)**		
≥33months	251	32.7
≤32months	516	67.3
**Sources of knowledge (n = 767)**		
Health care provider	584	76.2
Mass media	31	4.0
Family and friends	144	18.8
Social media	8	1.0
**Which birth interval has health advantage?**		
Below 3yars	92	10.5
3–5 years	592	67.2
Above 5 years	177	20.1
I do not know	19	2.2
**If you said 3–5 years interval have health advantage, for whom? (n = 1,514)**		
mother’s health	515	58.5
newborns and child health	565	64.2
Next child’s health	434	49.3
**Which birth interval has health disadvantage? (n = 1586)**		
Below 3 years	757	86.0
3 to 5 years	810	92.0
Above 5 years	19	2.2
**Practice of birth spacing is good (n = 880)**		
No	15	1.7
Yes	865	98.3
**Know methods of birth spacing? (n = 880)**		
No	28	3.2
Yes	852	96.8

**Table 4:** Attitude of study participants towards short birth interval in Dembecha district, North West Ethiopia, 2019 (n = 880).

Variables	Frequency	Percent (%)
**I want to practice birth spacing**		
Strongly agree	274	31.1
Agree	560	63.7
No opinion	5	0.6
Disagree	39	4.4
Strongly disagree	2	0.2
**Minimum 3 years is essential for birth interval**		
Strongly agree	264	30.0
Agree	559	63.5
No opinion	8	0.9
Disagree	43	4.9
Strongly disagree	6	0.7
**Birth spacing needs husband’s willingness**		
Strongly agree	316	35.9
Agree	537	61.0
No opinion	11	1.3
Disagree	11	1.3
Strongly disagree	5	0.6
**Short birth interval can be harmful to newborn**		
Strongly agree	267	30.3
Agree	512	58.2
No opinion	52	5.9
Disagree	47	5.3
Strongly disagree	2	0.2
**Short birth interval affects health of mother**		
Strongly agree	299	34.0
Agree	452	51.4
No opinion	74	8.4
Disagree	53	6.0
Strongly disagree	2	0.2
**Short birth interval affects health of father**		
Strongly agree	246	28.0
Agree	453	51.5
No opinion	81	9.2
Disagree	92	10.5
Strongly disagree	8	0.9
**Women should have delivered until they get male child**		
Strongly agree	26	3.0
Agree	127	14.4
No opinion	68	7.7
Disagree	506	57.5
Strongly disagree	153	17.4
**Short birth interval proves women fertility**		
Strongly agree	15	1.7
Agree	71	8.1
No opinion	107	12.2
Disagree	541	61.5
Strongly disagree	146	16.6
**No need to limit birth, it against will of God**		
Strongly agree	19	2.2
Agree	163	18.5
No opinion	103	11.7
Disagree	495	56.3
Strongly disagree	100	11.4
**Parents who have few children are rich**		
Strongly agree	162	18.4
Agree	413	46.9
No opinion	73	8.3
Disagree	193	21.9
Strongly disagree	39	4.4
**Women have equal responsibility as men for birth planning**		
Strongly agree	154	17.5
Agree	617	70.1
No opinion	18	2.0
Disagree	80	9.1
Strongly disagree	11	1.3
**Having few children may create a feeling of economically in secure**		
Strongly agree	41	4.7
Agree	227	25.8
No opinion	101	11.5
Disagree	461	52.4
Strongly disagree	50	5.7
**Many children can lead to tiredness and psychological problem**		
Strongly agree	197	22.4
Agree	515	58.5
No opinion	69	7.8
Disagree	93	10.6
Strongly disagree	6	0.7
**Short births may lead to health problems**		
Strongly agree	238	27.0
Agree	599	68.1
No opinion	21	2.4
Disagree	17	1.9
Strongly disagree	5	0.6
**Having many children makes family less happy**		
Strongly agree	146	16.6
Agree	505	57.4
No opinion	63	7.2
Disagree	157	17.8
Strongly disagree	9	1.0
**Having many children reduces the quality of care provided to them**		
Strongly agree	197	22.4
Agree	571	64.9
No opinion	39	4.4
Disagree	59	6.7
Strongly disagree	14	1.6
**Child birth until you get female child is good**		
Strongly agree	2	0.2
Agree	119	13.5
No opinion	62	7.0
Disagree	464	52.7
Strongly disagree	233	26.5

### Prevalence of short birth interval

The prevalence of short birth intervals was found to be 382 (43.4%) with 95% CI:(40.2,46.9) and the median birth interval of this study was 36 months with an IQR of 8 months.

### Factors associated with short birth intervals

On bivariable analysis, maternal age, maternal occupation, husband education, residence, wealth index, duration of breastfeeding the index child, age at first marriage, maternal age at delivery of index child, family planning use before conceiving last child, the survival of index child and knowledge of the study participants were significantly associated with a short birth interval at a p-value <0.2. However, husband’s education, wealth index, residence, age at first marriage, and non-use of contraceptives were the factors associated with the outcome variable in multivariable logistic regression.

Accordingly, women whose husbands could only be able to read and write were 2.81 times more likely to have short birth intervals compared to those women whose husbands completed college education and above (AOR = 2.81;95% CI:1.01, 7.85). Likewise, being in the lowest wealth quartile was 3.75 (AOR = 3.75;95% CI:2.35, 5.97) times more likely to have a short birth interval as compared with those in the highest wealth quartile.

This study revealed that women who first married at the age of 15–17 years were 1.65 times more likely to have a short birth interval compared to women who married at the age of 18 years and above (AOR = 1.65; 95% CI:1.18, 2.31). Similarly, the odds of having a short birth interval was 3.2 times higher among urban residences (AOR = 3.2;95% CI: 1.62, 6.33) compared with rural residences. Moreover, women who did not use contraceptives were 8.78 times more likely to have short birth intervals as compared to women who used contraceptives (AOR = 8.78; 95% CI; 6.18,12.47) (Table 5).

**Table 5:** Bivariable and multivariable analysis of factors associated with short birth intervals in Dembecha district, Northwest Ethiopia, 2019 (n = 880).

Variables	Short birth intervals		COR (95%CI)	AOR (95%CI)
	Yes	No		
**Maternal age groups**				
15–24	18	18	0.89 (0.42, 1.85)	1.07 (0.46, 2.48
25–29	93	109	0.76 (0.49, 1.17)	1.54 (0.92, 2.57)
30–34	95	144	0.58 (0.38, 0.89)	0.69 (0.42, 1.13)
35–39	102	161	0.56 (0.37, 0.85)	0.72 (0.44, 1.18)
40–49	74	66	1	1
**Maternal occupation**				
Employed	17	24	1	1
House wife	88	75	1.65 (0.82, 3.31)	0.58 (0.21, 1.63)
Merchant	20	12	2.35 (0.91, 6.07)	1.02 (0.30, 3.43)
Student	5	2	3.52 (0.61, 20.38)	1.24 (0.16, 9.41)
Farmer	243	381	0.90 (0.47, 1.71)	0.43 (0.15, 1.19)
Daily laborer	9	4	3.17 (0.83, 12.03)	1.07 (0.15, 7.47)
**Residence**				
Rural	335	469	1	1
Urban	47	29	2.27 (1.39, 3.68)	**3.20 (1.62, 6.33**) *
**Husband education**				
Unable to read and write	51	94	1.08 (.45, 2.58)	1.95 (0.66, 5.77)
Able to read and write	251	272	1.85(.81, 4.2)	**2.81 (1.01, 7.85)** *
Primary education	36	65	1.10 (.45, 2.71)	1.56 (.51, 4.71)
Secondary education	23	30	1.53 (.58, 4.03)	1.46 (.47, 4.57)
Collage and above	9	18	1	
**Wealth index quartile**				
Lowest	96	76	1.88 (1.30, 2.72)	**3.75 (2.35,5.97)** **
Second	77	103	1.11 (.77, 1.60)	1.27 (.80,2.02)
Third	68	109	.92 (.64,1.35)	.89 (.56,1.41)
Highest	141	210	1	1
**Maternal age at index child**				
15–19	13	8	1.16 (0.38, 3.49)	1.00 (0.22, 4.46)
20–29	189	276	0.48 (0.24, 0.97)	0.66 (0.25, 1.76)
30–39	159	199	0.57 (0.28, 1.14)	0.82 (0.34, 1.97)
40–49	21	15	1	1
**Duration of breastfeeding**				
0-12months	42	65	0.77 (0.50, 1.17)	0.73 (0.38, 1.38)
13-23months	69	105	0.78 (0.55, 1.10)	1.04 (0.62, 1.76)
≥24months	259	309	1	1
**Survival of index child**				
Yes	374	480	1	1
No	8	18	.57 (.24, 1.32)	0.45 (0.16, 1,25)
**Contraceptive use**				
Yes	165	409	1	1
No	217	89	6.04 (4.45, 8.20)	**8.78 (6.18, 12.47**) **
**Age at first marriage**				
15-17years	217	253	1.29 (0.98, 1.70)	**1.65 (1.18, 2.31**) *
≥18years	155	234	1	1
**Knowledge of participants**				
Knowledgeable	246	342	1	1
Not knowledgeable	136	156	1.21 (.91, 1.60)	0.94 (0.66, 1.34)

Note: COR-crude odds ratio, AOR-adjusted odds ratio, CI-confidence interval, 1-reference category

*p<0.05

**p<0.001.

## Discussion

This study tried to assess the prevalence and factors associated with short birth intervals among women who gave birth in the last three years in Dembecha district.

In our study, about 43.4% of women had short birth intervals (95% CI:40.2, 46.9). This finding is comparable with the study conducted in Debre Markos town which is 40.9% [21]. But, the finding of this study was higher than other studies done in Tigray, Ethiopia (23.3%) [9], Iran (28.5%) [22], and Bangladesh (24.6%) [19]. The possible reason for the discrepancy could be the difference in the selection criteria of the study participants and socio-cultural differences. The study in Tigray excluded mothers who had twin deliveries, preterm birth, and who didn’t have birth certificate. Nevertheless, in this study, we excluded only mothers who had abortions between the last two deliveries. This might have increased the prevalence of short birth intervals in this study. In the case of the studies carried out in Iran and Bangladesh, there is a socio-economic difference from our study. The women in the aforementioned studies, in Iran and Bangladesh, were mostly educated and residing in urban areas contrary to our study, where the majority of the respondents were uneducated and rural residents.

This study’s result was lower than the result of studies conducted in Hosanna (57.6%) [20], Oromia Illubabor zone (51.2%) [6], and Jimma (59.9%) [23]. This variation might be due to the difference in defining criteria of short birth intervals. The previous studies used 36 months as a criterion to define short birth intervals opposite to the current study which used 33 months as per the WHO recommendation, which might have relatively decreased the prevalence.

In this study, women whose husbands could read and write but had not completed primary or above level of education were 2.81 times more likely to have short birth intervals after the index child compared to those women whose husbands completed their college education and above. This finding is supported by a study conducted in Dabat district [5]. The possible explanation could be the fact that most of the decisions on the utilization of maternal health care services are made mainly by the husbands. If the husbands hadn’t had formal education, they might not have known the consequences of short birth intervals and encouraged their wives to have many children within a short period [24].

Women residing in an urban area were 3.2 times more likely to have short birth intervals than women residing in rural areas. The possible reason might be that most rural women breastfeed their newborns exclusively for a longer period than urban women do. Hence, they might benefit from lactational amenorrhea. This reasoning is supported by a study conducted in Niger, which suggested that the median duration of exclusive breastfeeding was only one week amongst urban women contrary to two months among rural women [25].

Women from the families within the lowest quartile were 3.75 times more likely to have a short birth interval than their highest quartile counterparts. This finding is in line with the studies conducted in Arba Minch district in Ethiopia, Bangladesh, and Nepal [1,19,26]. The possible reason might be that children born from poor households are assumed to be a source of income for the entire household thereby leading to short birth intervals.

This study showed that women who didn’t use postpartum contraceptives after the index child were 8.78 times more likely to have short birth intervals compared to contraceptive users. This finding is supported by studies conducted in Oromia, south pastoral community, and Dabat district [5,6,27].

The age of mothers at first marriage had a statistically significant association with short birth intervals. This study revealed that women who married at the age of 15–17 years were 1.65 times more likely to have short birth intervals than women who married at the age of 18 years or above. Similar findings were found in the studies conducted in Bangladesh and Nepal [19,26]. The reason might be that women who married at the age of 18 years and above would have a better opportunity for getting information, and education. Besides, having more power in making decisions on future fertility and growing older might have resulted a decline in women’s fecundity, which increases the time it takes to become pregnant.

### Limitations of the study

This study acknowledged some important possible limitations that should be considered when interpreting the results. As some of the questions are sensitive, social desirability bias might be present, and since they responded to previous birth interval periods, recall bias might be introduced. There might be also a social desirability bias on the declaration of the sex of their child. Lastly, as the reported sex ratio is too low and the percentage of children who died is likely also too low, there could be an omission of male children who died.

## Conclusion

Short birth interval practice is high in the study area. Factors like, women’s husbands who can only be able to read and write compared to more education, non-use of contraceptives, women found in the lowest wealth quartile, first marriage at 15–17 years, and urban residence were significantly associated with short birth intervals. Therefore, actions should be taken to increase family planning utilization, avoid early marriage, and strengthen paternal education to reduce short birth intervals.

## Supporting information

S1 Table Amharic and English version of the questionnaire. (DOCX)

S1 File SPSS dataset. (SAV)

S1 Appendix (DOCX)

## Abbreviations

ANC: antenatal care
AOR: adjusted odds ratio
CI: confidence interval
IQR: inter quartile range
SPSS: Statistical package of social science
WHO: world health organization
