# Supplementary material for: Short birth interval and associated factors among women who gave birth in the last three years in Dembecha district, Northwest Ethiopia
Source: PLoS One. 2022 Aug 23;17(8):e0272612. doi: 10.1371/journal.pone.0272612 (PMC9398008; doi:10.1371/journal.pone.0272612)
Supplement: S1 Table — (DOCX) [file pone.0272612.s001.docx]

**Annex IV: Questionnaire English Version**

Instruction: please encircle the number listed before the option to indicate your response and fill the blank for without option.

Code No _________

| **Part I**: **socio- demographic characteristics** | | | | | | | | | | | | | | | | | | | | | | | | | | |
| --- | --- | --- | --- | --- | --- | --- | --- | --- | --- | --- | --- | --- | --- | --- | --- | --- | --- | --- | --- | --- | --- | --- | --- | --- | --- | --- |
| \| SN \|  \|  \|  \| \| --- \| --- \| --- \| --- \| | | | | | | Questions | | | | | | | | | Answers/choices | | | Skip | | | | | | | | |
| 101 | | | | | | How old are you? | | | | | | | | | ------------completed years | | |  | | | | | | | | |
| 102 | | | | | | What is your marital status? | | | | | | | | | 1. Single  2. Married  3. Divorced  4. Widowed | | |  | | | | | | | | |
| 103 | | | | | | What is your religion? | | | | | | | | | 1. Orthodox  2. Protestant  3. Muslim  4. Catholic  5.Others(specify)_____ | | |  | | | | | | | | |
| 104 | | | | | | What is your educational level? | | | | | | | | | 1. unable to read and write  2. Able to read and write  3.secondary education  4. collage and above | | |  | | | | | | | | |
| 105 | | | | | | What is your primary Occupation? | | | | | | | | | 1.Employed (governmental /Private )  2. Housewife  3. Merchant  4. Student  5. Farmer  6. Daily laborer  7.Others______ | | |  | | | | | | | | |
| 106 | | | | | | Where is your residence? | | | | | | | | | 1.urban 2.rural | | |  | | | | | | | | |
| 107 | | | | | | What is your husband's education level? | | | | | | | | | 1.unable to read and write  2. Able to read and write  3.secondary education  4.collage and above | | |  | | | | | | | | |
| 108 | | | | | | What is your husband’s primary occupation? | | | | | | | | | 1.Employed (governmental /Private)  2. Merchant  3. Student  4. Farmer  5. Daily laborer  6.Others(Specify)___ | | |  | | | | | | | | |
| **Part II**: **Reproductive History of the respondents** | | | | | | | | | | | | | | | | | | | | | | | | | | |
| 201 | | | | | | What was your age at first marriage? | | | | | | | | ………..completed years | | | | | |  | | | | | | |
| 202 | | | | | | How many times did you deliver totaly? | | | | | | | | ………….times | | | | | |  | | | | | | |
| 203 | | | | | | | How many of your own living children do you have currently? | | | | | | | | Male____ Females_____ | | | |  | | | | | | | |
| 204 | | | | | | | How old were you when you deliver your index child? | | | | | | | | ----------years | | | |  | | | | | | | |
| 205 | | | | | | | Did you have antenatal care follow-up for your index child? | | | | | | | | 1.Yes 2.No | | | | If say 2 go to Q207 | | | | | | | |
| 206 | | | | | | | If your answer for Q;205 is yes how many times did you visit the ANC clinic? | | | | | | | | 1.once 2.twice  3.three times 4.four and above | | | |  | | | | | | | |
| 207 | | | | | | | During ANC follow up did you get any information regarding short birth interval? | | | | | | | | 1.yes  2.No | | | |  | | | | | | | |
| 208 | | | | | | | Where did you deliver your index child? | | | | | | | | 1.Health facility  2.Home  3.others------------- | | | |  | | | | | | | |
| 209 | | | | | | | What was the Sex of the index child? | | | | | | | | 1.Female 2.Male | | | |  | | | | | | | |
| 210 | | | | | | | How many neonate deliverdat index child? | | | | | | | | 1.Singleton  2.multiple delivery | | | |  | | | | | | | |
| 211 | | | | | | | How was the survival status of the index child currently? | | | | | | | | 1. Alive 2. Dead | | | |  | | | | | | | |
| 212 | | | | | | | Have you breast feed your index child? | | | | | | | | 1.yes 2.No | | | | If 2 go to 215 | | | | | | | |
| 213 | | | | | | | If yes for Q 212 How many months? | | | | | | | | ………..months | | | |  | | | | | | | |
| 214 | | | | | | | Was the last delivery planned? | | | | | | | | 1.Yes 2.No | | | |  | | | | | | | |
| 215 | | | | | | | After you delivered your index child did you want to have more children? | | | | | | | | 1.Yes 2.No | | | | If 2 go toQ217 | | | | | | | |
| 216 | | | | | | | If yes to Q215; in what period you want to deliver? | | | | | | | | 1.less than 24months  2.24-35months  3.36 months and above | | | |  | | | | | | | |
| 217 | | | | | | | Who is the ultimate decision-maker regarding birth spacing? | | | | | | | | 1.Self  2.Husband  3.Both | | | |  | | | | | | | |
| 218 | | | | | | | How was the belief of the husband regarding birth spacing? | | | | | | | | 1.Supports  2.Opposes  3.I don’t know | | | |  | | | | | | | |
| 219 | | | | | | | What was the length of the birth interval between the index child and the last child? | | | | | | | | ------------months | | | |  | | | | | | | |
| 220 | | | | | | | Before you become pregnant with the last child, did you used contraceptives? | | | | | | | | 1.yes 2.No | | | |  | | | | | | | |
| 221 | | | | | | | If your answer to Q;220 is yes, what was the aim of using this contraceptive? | | | | | | | | 1.birth spacing  2. limiting birth  3.other specify---------- | | | |  | | | | | | | |
| 222 | | | | | | | Did you communicate with your husband about the number children you will have? | | | | | | | | 1.yes 2.No | | | |  | | | | | | | |
| **Part III: Knowledge of women about Birth spacing** | | | | | | | | | | | | | | | | | | | | | | | | | | |
| 301 | | | | | Have you ever heard about short birth interval between two consecutive births? | | | | | | | | 1. Yes  2. No | | | | | | | | If 2 go toQ 303 | | | | | |
| 302 | | | | | If your answer to Q301 is yes, what is the number of years between two consecutive births called short birth interval? | | | | | | | | ---------years | | | | | | | |  | | | | | |
| 303 | | | | | Where did you get the information? | | | | | | | | 1.Health Facilities  2.Mass media  3.Family and friends  4 .social media | | | | | | | |  | | | | | |
| 304 | | | | | Which birth interval do you think have health advantage? | | | | | | | | 1. Below 3 years  2. 3 to 5 years  3. Above 5 years  4. I don’t know | | | | | | | | If not no .2 go to  306 | | | | | |
| 305 | | | | | If your answer to Q304 is 3-5 years, for whom do you think to have a health advantage?  (More than one answer possible) | | | | | | | | 1. mother’s health  2. newborns and childhealth  3.Next child’s health  4.Others(specify)______ | | | | | | | |  | | | | | |
| 306 | | | | | Which birth interval has health disadvantages?  (More than one answer possible) | | | | | | | | 1. Below 3 years  2. 3 to 5 years  3. Above 5 years  4. I don’t know | | | | | | | |  | | | | | |
| 307 | | | | | Birth spacing being a good practice | | | | | | | | 1.yes  2.No  3. no idea | | | | | | | |  | | | | | |
| 308 | | | | | Do you know birth spacing methods? | | | | | | | | 1.yes 2.No | | | | | | | |  | | | | | |
| **Part V: Attitude of respondents about birth spacing** | | | | | | | | | | | | | | | | | | | | | | | | | | |
| 401 | | | | | | | | | | I want to practice birth spacing | | | | | | | 1.Strongly agree  2. Agree  3.No opinion  4.Disagree  5.Strongly disagree | | | | | |  | | | |
| 402 | | | | | | | | | | I think minimum 3years is essential for birth spacing | | | | | | | 1.Strongly agree  2. Agree  3.No opinion  4.Disagree  5.Strongly disagree | | | | | |  | | | |
| 403 | | | | | | | | | | Need a husband’s willingness for birth spacing | | | | | | | 1.Strongly agree  2. Agree  3.No opinion  4.Disagree  5.Strongly disagree | | | | | |  | | | |
| 404 | | | | | | | | | | Having short birth interval can be harmful to the health of the newborn and the preceding child | | | | | | | 1.Strongly agree  2. Agree  3.No opinion  4.Disagree  5.Strongly disagree | | | | | |  | | | |
| 405 | | | | | | | | | | Having short birth interval can be harmful to the physical and mental health of the mother | | | | | | | 1.Strongly agree  2. Agree  3.No opinion  4.Disagree  5.Strongly disagree | | | | | |  | | | |
| 406 | | | | | | | | | | Having short birth interval can be harmful to the physical and mental health of the father | | | | | | | 1.Strongly agree  2. Agree  3.No opinion  4.Disagree  5.Strongly disagree | | | | | |  | | | |
| 407 | | | | | | | | | | \| The family that has all girls should keep having children until they have at least one boy \| \| --- \| | | | | | | | 1.Strongly agree  2. Agree  3.No opinion  4.Disagree  5.Strongly disagree | | | | | |  | | | |
| 408 | | | | | | | | | | \| Having short birth interval is important for the woman to prove her fertility \| \| --- \| | | | | | | | 1.Strongly agree  2. Agree  3.No opinion  4.Disagree  5.Strongly disagree | | | | | |  | | | |
| 409 | | | | | | | | | | \| Women should not limit the number of children they should have as it is against the will of God? \| \| --- \| | | | | | | | 1.Strongly agree  2. Agree  3.No opinion  4.Disagree  5.Strongly disagree | | | | | |  | | | |
| 410 | | | | | | | | | | \| Parents with fewer children have better economic status? \| \| --- \| | | | | | | | 1.Strongly agree  2. Agree  3.No opinion  4.Disagree  5.Strongly disagree | | | | | |  | | | |
| 411 | | | | | | | | | | \| Women are as much responsible for planning births as men? \| \| --- \| | | | | | | | 1.Strongly agree  2. Agree  3.No opinion  4.Disagree  5.Strongly disagree | | | | | |  | | | |
| 412 | | | | | | | | | | \| Having a few children may cause a person to feel economically insecure in old age? \| \| --- \| | | | | | | | 1.Strongly agree  2. Agree  3.No opinion  4.Disagree  5.Strongly disagree | | | | | |  | | | |
| 413 | | | | | | | | | | A large number of children can lead to tiredness and psychological problems for parents? | | | | | | | 1.Strongly agree  2. Agree  3.No opinion  4.Disagree  5.Strongly disagree | | | | | |  | | | |
| 414 | | | | | | | | | | \| Frequent births may lead to health problems \| \| --- \| | | | | | | | 1.Strongly agree  2. Agree  3.No opinion  4.Disagree  5.Strongly disagree | | | | | |  | | | |
| 415 | | | | | | | | | \| Compared with small families large families are less happy \| \| --- \| | | | | | | | | 1.Strongly agree  2. Agree  3.No opinion  4.Disagree  5.Strongly disagree | | | | | |  | | | |
| 416 | | | | | | | | | \| Having short birth interval affects the quality of health care provided to children \| \| --- \| | | | | | | | | 1.Strongly agree  2. Agree  3.No opinion  4.Disagree  5.Strongly disagree | | | | | |  | | | |
| 417 | | | | | | | | | \| The family that has all boys should keep having children until they have at least one girl. \| \| --- \| | | | | | | | | 1.Strongly agree  2. Agree  3.No opinion  4.Disagree  5.Strongly disagree | | | | | |  | | | |
| **Part V: house hold wealth status of respondents** | | | | | | | | | | | | | | | | | | | | | | | | | | |
| 501 | | | What is the main source of drinking water for the members of your household? | | | | | | | | | 1. Piped water  2. Protected well  3. unprotected well  4. protected spring  5. unprotected spring  6. surface water (River, pond)  7.Others(specify)_________ | | | | | | | | | | |  | | | |
| 502 | | | Do you have toilet? | | | | | | | | | 1.yes 2.No | | | | | | | | | | |  | | | |
| 503 | | | Is the toilet private? | | | | | | | | | 1.yes 2.No | | | | | | | | | | |  | | | |
| 504 | | | What kind of toilet facility do members of your household usually use? | | | | | | | | | 1. Flush toilet  2. Ventilated improved pit latrine (VIP)  3. Pit latrine with slab  4. Pit latrine without slab/ open pit  5. others (specify __ | | | | | | | | | | |  | | | |
| 505 | | | Which of the following assets do you have in your household?  (more than one answer’s possible) | | | | | | | | | 1) Electricity  2) Watch  3) Radio  4) Television  5) Mobile telephone  6) Non-mobile telephone  7) Refrigerator  8) Table  9) Chair  10) Sofa  11) Bed  12) Cabinet  13) Car  14) Motorcycle  15) Animal-drawn cart  16) Bicycle  17)Others(specify)______ | | | | | | | | | | |  | | | |
| 506 | | | What type of fuel do you use manily for cooking in your household?  *(Circle only one answer )* | | | | | | | | | 1) Electricity  2) Kerosene  3) Charcoal  4) Wood  5) Animal dung  6) Others (specify | | | | | | | | | | |  | | | |
| 507 | | | Owner ship of the house | | | | | | | | | 1. Private 2. Rented from individual   3.Others(specify)_______ | | | | | | | | | | |  | | | |
| 508 | | | Number of rooms in the household used for sleeping | | | | | | | | | -----------------in number | | | | | | | | | | |  | | | |
| 509 | | | Is the cooking usually done in the house, in a separate building, or outdoors? | | | | | | | | | 1. In a separate room used as kitchen 2. Elsewhere in the house 3. In a separate building 4. Outdoors   5.Other (specify)_____ | | | | | | | | | | |  | | | |
| 510 | | | What is the material of your house floor? (observation)  *Circle only one answer* | | | | | | | | | 1) Ceramic tiles  2) Cement/bricks  3) Wood  4) Mud  5) Other (specify)________ | | | | | | | | | | |  | | | |
| 511 | | | What is the material of the roof of your house (observation)  *Circle only one answer* | | | | | | | | | 1) Corrugated iron sheet  2) Cement  3) Wood  4) Plastic sheets  5) Thatch/leaf  6)Other(specify)____________ | | | | | | | | | | |  | | | |
| 512 | | | What is the material of the walls of your house (observation)  *Circle only one answer* | | | | | | | | | 1) Wood with mud  2) Wood with Cement  3) Stone with cement  4) Other[specify]______ | | | | | | | | | | |  | | | |
| 513 | | | What is the material of the Windows?  (Observation) | | | | | | | | | 1.Shutters  2.Glass  3.Screens  5.Others(specify) | | | | | | | | | | |  | | | |
| 514 | | | What is the primary source of income for this household?  *Circle only one answer* | | | | | | | | | 1. Farming2. Livestock  3. Employment/salary  4. Petty trading (including sale of fire-wood, charcoal, grass)  5. Daily labor  6.Others(specify)___________ | | | | | | | | | | |  | | | |
| 515 | | | Does any member of the household own any land that can be used for agriculture? | | | | | | | | | 1.Yes  2.No | | | | | | | | | | | If 2 go to No518 | | | |
| 516 | | | If yes Ownership of the agricultural land | | | | | | | | | 1.Own,in hectares____  2.Rent, in hectares____ | | | | | | | | | | |  | | | |
| 517 | | | On average How many farm lands do you have? | | | | | | | | | -------------hectares | | | | | | | | | | |  | | | |
| 518 | | | Annual total agricultural products (includes all items) | | | | | | | | | ----------kuntals | | | | | | | | | | |  | | | |
| 519 | | | Does this household own any livestock, herds, or farm animals? | | | | | | | | | 1.Yes  2.No | | | | | | | | | | | If no 2 go to no 522 | | | |
| 520 | | | Do you have your own animals? | | | | | | | | | 1.yes 2.No | | | | | | | | | | |  | | | |
| 521 | | | How many of the following animals does this household own?  [Multiple answers is possible] | | | | | | | | | 1. Cows___  2. Oxen/bulls_____  3.Horses  4.Donkeys----------------5.Mules_______  6. Sheep-------------  7.Goats____________  8.Beehives  9.Others(specify)___________ | | | | | | | | | | |  | | | |
| 522 | | | Does any member of this household have a bank account? | | | | | | | | | 1.yes  2.No | | | | | | | | | | |  | | | |
| Thank you!    **Annex VI: የአማረኛ መጠይቅ**  መመሪያ፡ከተዘረዘሩት ምርጫዎች መልስ የያዘዉን ቁጥር በማክበብ እና ምርጫ ለሌላቸዉ ጥያቄዎች ክፍት ቦታዉን በመሙላት ይመለሱ።  መለያቁጥር------------------------- | | | | | | | | | | | | | | | | | | | | | | | | | | |
| ተ.ቁ | | | ጥያቄዎች | | | | | | ምላሽ | | | | | | | | | | | | | | | ይለፉት | | |
| **ክፍል- I ማህበራዊነክ መረጃዎችን በተመለከተ የሚሰበሰብ** | | | | | | | | | | | | | | | | | | | | | | | | | | |
| 101 | | | እድሜዎት ስንት ነዉ? | | | | | | --------------ዓመት | | | | | | | | | | | | | | |  | | |
| 102 | | | የጋብቻ ሁኔታዎ ምንድን ነዉ? | | | | | | 1.ያላገባች 3. የፈታች  2.ያገባች 4.የሞተባት | | | | | | | | | | | | | | |  | | |
| 103 | | | ሐይማኖትዎ ምንድን ነዉ? | | | | | | 1.ኦርቶዶክስ 2.ሙስሊም  3.ፕሮቴስታንት 4.ካቶሊክ  5.ሌላይግለፁ------- | | | | | | | | | | | | | | |  | | |
| 104 | | | የትምህርትደረጃዎትስንትነዉ? | | | | | | 1.ማንበብ እና መጻፍ የማይችል  2.ማንበብ እና መጻፍ የሚችል  3.የመጀመሪያ ደረጃ ትምህርት  4.ሁለተኛ ደርጃ ትምህርት  5.ኮሌጅ እና ከዚያ በላይ | | | | | | | | | | | | | | |  | | |
| 105 | | | የስራ ሁኔታዎት ምንድን ነዉ? | | | | | | \| 1.ተቀጣሪ (የመንግስት/የግል) \| \| --- \|   2.የቤትእመቤት 3.ነጋዴ  4. ተማሪ 5.አርሶአደር  6. የቀንሰራተኛ 7. ሌላ(ይገለፅ)--- | | | | | | | | | | | | | | |  | | |
| 106 | | | መኖርያቤትዎየትነዉ? | | | | | | 1. ገጠር 2.ከተማ | | | | | | | | | | | | | | |  | | |
| 107 | | | የትዳር ጓደኛዎ የትምህርት ደረጃ ምንድረስ ነዉ ? | | | | | | 1.ማንበብ እና መጻፍየማይችል  2.ማንበብ እና መጻፍየሚችል  3. የመጀመሪያደረጃትምህርት  4.ሁለተኛ ደርጃ ትምህርት  5.ኮሌጅ እና ከዚያ በላይ | | | | | | | | | | | | | | | ካላገቡ ወደ ጥ.ቁ 201 ይለፉ | | |
| 108 | | | የትዳር ጓደኛዎ ስራ ምንድን ነው? | | | | | | 1.የግልተቀጣሪ(የመንግስትተቀጣሪ)  2.ነጋዴ  3.ተማሪ 4.አርሶአደር  5.የቀንሠራተኛ 6.ሌላይግለፁ-- | | | | | | | | | | | | | | |  | | |
| **ክፍል II: የጥናቱ ተሳታፊዎችን ስነ-ተዋልዶ በተመለከተ የሚሰበሰብ መረጃ** | | | | | | | | | | | | | | | | | | | | | | | | | | |
| 201 | | መጀመሪያሽን ስታገቢ ዕድሜሽ ስንት ነበር? | | | | | | | | | | | | | | -----------ዐመት | | | | | | |  | | | |
| 202 | | በአጠቃላይ ስንት ጊዜ ወልደዋል ? | | | | | | | | | | | | | | ----------------ጊዜ | | | | | | |  | | | |
| 203 | | አሁን በህይዎት ያሉ ስንት ልጆች አሉዎት?   \|  \| \| --- \| | | | | | | | | | | | | | | ወንድ____ ሴት___ | | | | | | |  | | | |
| 204 | | ከአሁኑ ልጅዎ በፊት ያለዉን ልጅ የወለዱት በስንት አመትዎ ነው? | | | | | | | | | | | | | | ---------------ዓመት | | | | | | |  | | | |
| 205 | | ከአሁኑ ልጅዎ በፊት ያለዉን ልጅዎን እንዳረገዙ የእርግዝና ክትትል አድርገው ነበር ? | | | | | | | | | | | | | | 1.አዎ 2.አላደረኩም | | | | | | | መልስዎ 2ከሆነ ወደ 208ይለፉ | | | |
| 206 | | ለ206 መልስዎ አዎ ከሆነ ስንት ጊዜ የእርግዝና ክትትል አድርገዋል? | | | | | | | | | | | | | | -----------ጊዜ | | | | | | |  | | | |
| 207 | | የእርግዝና ክትትል በሚያደርጉበት ወቅት አራርቆ ስለመውለድ ትምህርት አግኝተዋል? | | | | | | | | | | | | | | 1.አዎ 2 .አላገኘሁም | | | | | | |  | | | |
| 208 | | ከአሁኑ ልጅዎ በፊት ያለዉን ልጅዎን የትነው የወለዱት ? | | | | | | | | | | | | | | 1.ጤናተቋም  2.ቤት  3.ሌላካለይግለጹ…….. | | | | | | |  | | | |
| 209 | | ከአሁኑ ልጅዎ በፊት ያለዉል ጅዎ ፆታ ምን ነበር? | | | | | | | | | | | | | | 1.ሴት 2.ወንድ | | | | | | |  | | | |
| 210 | | ከአሁኑ እርግዝናዎ በፊት በነበረዉ እርግዝናዎ ስንት ልጆችን ተገላገሉ/ወለዱ? | | | | | | | | | | | | | | 1.አንድ ብቻ  2.ሁለት እና ከዚያ በላይ | | | | | | |  | | | |
| 211 | | ከአሁኑ ልጅ በፊት ያለዉ ልጅዎ አሁን በህይዎት አለ? | | | | | | | | | | | | | | 1. በህይዎት አለ 2.የለም | | | | | | |  | | | |
| 212 | | ከአሁኑ ልጅዎ በፊት ያለዉን ልጅዎን ጡት ያጠቡ ነበር | | | | | | | | | | | | | | 1.አዎ 2.የለም | | | | | | | መልስዎ 2 ከሆነወደ2014ይለፉ | | | |
| 213 | | መልስዎ አዎ ከሆነ ለጥያቄ ቁ.212 ለምን ያህል ወራት ጡት አጠቡ? | | | | | | | | | | | | | | ------ወራት | | | | | | |  | | | |
| 214 | | ከአሁን በፊት ያለው እርግዝና የታቀደ ነበር? | | | | | | | | | | | | | | 1.አዎ 2. አይደለም | | | | | | |  | | | |
| 215 | | ከአሁኑ ልጅ በፊት ያለዉን ልጅዎን ከወለዱ በኃላ ተጨማሪ ልጅ የመዉለድ ፍላጎት ነበረዎት? | | | | | | | | | | | | | | 1. አዎ  2.አልነበረኝም | | | | | | | መልስዎ 2ከሆነወደ 217  ይለፉ | | | |
| 216 | | ለጥያቄ ቁ.215 መልስዎ አዎ ከሆነ በምን ያህል ጊዜ ልጅ እንዲኖርዎት ነበር የፈለጉት? | | | | | | | | | | | | | | 1. ከ24 ወራትበታች  2. 24-35 ወራት  3. 36 ወራትእና ከዛበላይ | | | | | | |  | | | |
| 217 | | እናንተ ቤተሰብ ውስጥ ስለአራርቆ መወለድ ወሳኙ ማነው ? | | | | | | | | | | | | | | 1.እኔ  2. ባለቤቴ  3.እኔናባለቤቴ  4.ሌላየቤተሰብአባል  5. ሌላ(ይገለፅ)---- | | | | | | |  | | | |
| 218 | | ባለቤትዎ ስለአራርቆ መወለድ ያላቸው አቋም ምን ነበር ? | | | | | | | | | | | | | | 1.ይደግፋል 2.ይቃወማል  3.አላቅም | | | | | | |  | | | |
| 219 | | በመጨረሻ ልጅዎና በእሱ/ሳ ቀጣይ ታላቅ ምን ያህል የእድሜ ልዩነት አለ ? | | | | | | | | | | | | | | -------------ወራት | | | | | | |  | | | |
| 220 | | የመጨረሻ ልጅሽን ከማርገዝሽ በፊት የእርግዝና መከላከያ ትጠቀሚ ነበር? | | | | | | | | | | | | | | 1.አዎ  2.አልጠቀመም | | | | | | |  | | | |
| 221 | | ለጥያቄቁ. 223፤መልስዎ አዎ ከሆነ አላማዉ ለምን ነበር? | | | | | | | | | | | | | | 1.አራርቆ ለመዉለድ  2.ልጅንለ መመጠን  3.ሌላ ካለይግለጹ----- | | | | | | |  | | | |
| 222 | | ምን ያህል ልጆች ሊኖርዎት እንደሚፈልጉ ከባለቤትዎ ጋር ተነጋግረው ያዉቃሉ? | | | | | | | | | | | | | | 1. አዎ 2.አላውቅም | | | | | | |  | | | |
| **ክፍል- III እናቶች ስለአቀራርቦ/ቶሎቶሎ መውለድ ያላቸውን እውቀት በተመለከተ** | | | | | | | | | | | | | | | | | | | | | | | | | | |
| 301 | | አቀራርቦ/ቶሎቶሎ መዉለድ ምን ማለት እንደሆነ ሰምተዉ ያቃሉ? | | | | | | | 1.አዎ 2. አላቅም | | | | | | | | | | | | | | | መልስዎ 2 ከሆነ ወደጥ.ቁ 4 ይለፉ | | |
| 302 | | ለጥያቄ ቁ.301 መልስዎ አዎ ከሆነ ከስንት ወራት በታች ነዉ? | | | | | | | --------------ወራት | | | | | | | | | | | | | | |  | | |
| 303 | | ይህንን እዉቀትከየትነዉያገኙት? | | | | | | | 1.ጤና ተቓም  2.ከመገናኛ ብዙሀን  3. ከቤተሰብ እና ጋደኛ  4.ሌላካለ-------- | | | | | | | | | | | | | | |  | | |
| 304 | | በሁለቱ ተከታታይ የወሊድ ጊዜ፤የትኛዉ የጊዜ ልዩነት ነዉ የጤና ጥቅም ያለዉ? | | | | | | | 1.ከ3 ዓመትበተታች  2. ከ3-5 ዓመት  3. ከ5 ዓመትበላይ  4. አላቅም | | | | | | | | | | | | | | | መልስዎ2 ካልሆነወደ 306 ይለፉ | | |
| 305 | | ጥያቄቁ.304 መልስዎ ከ3-5 ዐመት ከሆነ፤ለማን ነዉ? የጤና ጥቅም ያለዉ (ከአንድበላይመልስይቻላል) | | | | | | | 1.ለእናትዬዋ ጥቅም  2.ለጨቅላ ህፃናት እና ለልጆች  3.ለሚቀጥለዉ ልጅ ጤና  4.ሌላ ካለ ይግለፁ---- | | | | | | | | | | | | | | |  | | |
| 306 | | በሁለቱ ተከታታይ የወሊድ ጊዜ፤የትኛዉ የጊዜ ልዩነት ነዉ የጤና ችግር የሚያስከትለዉ? | | | | | | | 1.ከ3 ዓመትበተታች  2. ከ3-5 ዓመት  3. ከ5 ዓመትበላይ  4. አላቅም | | | | | | | | | | | | | | |  | | |
| 307 | | ልጅን አራርቆ መዉለድ ጥሩ ተግባርነ ነዉ? | | | | | | | 1.አዎ 2.አይደለም 3.ሀሳብ የለኝም | | | | | | | | | | | | | | |  | | |
| 308 | | አራርቆ ለመዉለድ የሚያገለግሉ መንገዶችን/ዘዴ ታቂያለሽ? | | | | | | | 1.አዎ 2.አላቅም | | | | | | | | | | | | | | |  | | |
| **ክፍል IV: አቀራርቦ/ቶሎቶሎ መዉለድን በተመለከተ ተሳታፊዎች ያላቸዉን አመለካከት ለማወቅየተዘጋጄ** | | | | | | | | | | | | | | | | | | | | | | | | | | |
| 401 | | አራርቆ መዉለድን መተግበር እፈልጋለዉ፡፡ | | | | | | | 1.በጣም እስማማለሁ  2. እስማማለሁ  3.አስተያየት የለኝም  4.አልስማማም  5.በጣም አልስማማም | | | | | | | | | | | | | |  | | | |
| 402 | | በእኔ አስተሳስብ በትንሹ 3 ዐመት አራርቆ መዉለድ በቂ ነዉ፡፡ | | | | | | | 1.በጣም እስማማለሁ  2. እስማማለሁ  3.አስተያየት የለኝም  4.አልስማማም  5.በጣም አልስማማም | | | | | | | | | | | | | |  | | | |
| 403 | | አራርቆ ለመዉለድ የባለቤቴ ፈቃደኝነት አስፈላጊ ነዉ፡፡ | | | | | | | 1.በጣም እስማማለሁ  2. እስማማለሁ  3.አስተያየት የለኝም  4.አልስማማም  5.በጣም አልስማማም | | | | | | | | | | | | | |  | | | |
| 404 | | ቶሎ ቶሎ መዉለድ ለሚወለዱት ልጆችና ለተወለዱት ልጆች የጤና ችግር ሊያመጣ ይችላል፡፡ | | | | | | | 1.በጣም እስማማለሁ  2. እስማማለሁ  3.አስተያየት የለኝም  4.አልስማማም  5.በጣም አልስማማም | | | | | | | | | | | | | |  | | | |
| 405 | | ቶሎ ቶሎ መዉለድ ለእናት አካላዊና ስነልቦናዊ ጤና ችግር ሊዳርግ ይችላል፡፡ | | | | | | | 1.በጣም እስማማለሁ  2. እስማማለሁ  3.አስተያየት የለኝም  4.አልስማማም  5.በጣም አልስማማም | | | | | | | | | | | | | |  | | | |
| 406 | | ቶሎ ቶሎ መዉለድ ለአባት አካላዊና ስነልቦናዊ ጤና ችግር ሊዳርግ ይችላል፡፡ | | | | | | | 1.በጣም እስማማለሁ  2. እስማማለሁ  3.አስተያየት የለኝም  4.አልስማማም  5.በጣም አልስማማም | | | | | | | | | | | | | |  | | | |
| 407 | | ሴት ልጆች ብቻ ያላቸው ቤተሰቦች ወንድ ልጅ እስኪያ ገኙ ድረስ መውለድ አለባቸው ፡፡ | | | | | | | 1.በጣም እስማማለሁ  2. እስማማለሁ  3.አስተያየት የለኝም  4.አልስማማም  5.በጣም አልስማማም | | | | | | | | | | | | | |  | | | |
| 408 | | ቶሎቶሎ መዉለድ የሴቶችን ወላድነት/የመውለድ አቅም ለማረጋገጥ አስፈላጊ ነው/ይጠቅማል ፡፡ | | | | | | | 1.በጣም እስማማለሁ  2. እስማማለሁ  3.አስተያየት የለኝም  4.አልስማማም  5.በጣም አልስማማም | | | | | | | | | | | | | |  | | | |
| 409 | | ከእግዚአብሄር ፈቃድ ውጭ ስለሆነ ሴቶች የሚወልዷቸውን ልጆች መመጠን የለባቸውም ፡፡ | | | | | | | 1.በጣም እስማማለሁ  2. እስማማለሁ  3.አስተያየት የለኝም  4.አልስማማም  5.በጣም አልስማማም | | | | | | | | | | | | | |  | | | |
| 410 | | ጥቂት ልጆች ያላቸው ቤተሰቦች የኢኮኖሚ ደረጃቸው የተሻለ ነው፡፡   \|  \| \| --- \| | | | | | | | 1.በጣም እስማማለሁ  2. እስማማለሁ  3.አስተያየት የለኝም  4.አልስማማም  5.በጣም አልስማማም | | | | | | | | | | | | | |  | | | |
| 411 | | ሴቶች ቤተሰብን ከመመጠን አካያ ከወንዶች እኩል ድርሻ አላቸው፡፡   \|  \| \| --- \| | | | | | | | 1.በጣም እስማማለሁ  2. እስማማለሁ  3.አስተያየት የለኝም  4.አልስማማም  5.በጣም አልስማማም | | | | | | | | | | | | | |  | | | |
| 412 | | ጥቂት ልጆች መኖራቸው ወላጆች በሚያረጁበት ጊዜ ኢኮኖሚያቸው የተረጋጋ እነዳይሆን ሊያደርግ ይችላል?   \|  \| \| --- \| | | | | | | | 1.በጣም እስማማለሁ  2. እስማማለሁ  3.አስተያየት የለኝም  4.አልስማማም  5.በጣም አልስማማም | | | | | | | | | | | | | |  | | | |
| 413 | | ብዙ ልጆች መኖራቸው ወላጆች ላይ  የስነልቦና ችግር ሊያስከትል ይችላል?   \|  \|  \| \| --- \| --- \| | | | | | | | 1.በጣም እስማማለሁ  2. እስማማለሁ  3.አስተያየ ትየለኝም  4.አልስማማም  5.በጣም አልስማማም | | | | | | | | | | | | | |  | | | |
| 414 | | ቶሎቶሎ መዉለድ ለጤና ችግር ሊያጋልጥ ይችላል ?   \|  \| \| --- \| | | | | | | | 1.በጣም እስማማለሁ  2. እስማማለሁ  3.አስተያየት የለኝም  4.አልስማማም  5.በጣም አልስማማም | | | | | | | | | | | | | |  | | | |
| 415 | | ብዙ ቤተሰብ ያላቸው ሰዎችትን ቤተሰብ ከሌላቸው ሲወዳደሩ ደስተኞች አይደሉም ?   \|  \| \| --- \| | | | | | | | .1.በጣም እስማማለሁ  2.እስማማለሁ  3.አስተያየት የለኝም  4.አልስማማም  5.በጣም አልስማማም | | | | | | | | | | | | | |  | | | |
| 416 | | ቶሎቶሎ መዉለድ ለልጆች የሚሰጠውን የጤና አገልግሎት ጥራት ይቀንሰዋል?   \|  \| \| --- \| | | | | | | | 1.በጣም እስማማለሁ  2. እስማማለሁ  3.አስተያየት የለኝም  4.አልስማማም  5.በጣም አልስማማም | | | | | | | | | | | | | |  | | | |
| 417 | | ወንድ ልጆች ብቻ ያላቸው ቤተሰቦች ሴት ልጅ እስኪያገኙ ድረስ መውለድ አለባቸው ? | | | | | | | 1.በጣም እስማማለሁ  2. እስማማለሁ  3.አስተያየት የለኝም  4.አልስማማም  5.በጣም አልስማማም | | | | | | | | | | | | | |  | | | |
| **ክፍል-V፡የቤተሰብ የሃብት መጠን የሚመለከት ጥያቄዎች** | | | | | | | | | | | | | | | | | | | | | | | | | | |
| 501 | | የቤተሰባችሁ አባላት የመጠጥ ዉሃ ከየት ነው የሚያገኙት? | | | | | | | | | 1.የቧነቧ ውሃ  2. ከተከለለ የጉድጓድ ውሃ  3.ካልተከለለ የጉድጓድ ውሃ  4. ከተከለለ ምንጭ  5.ካልተከለለ ምንጭ  6.የኩሬ ወይም የወንዝ ውሃ  7.ሌላ(ይገለፅ)____ | | | | | | | | | | |  | | | | |
| 502 | | ሽንት ቤት አላችሁ ? | | | | | | | | | 1. አዎ 2. የለንም | | | | | | | | | | | መልስዎ 2 ከሆነወደ  505 ይለፉ | | | | |
| 503 | | ሽንት ቤቱ የራሳችሁ ነው ? | | | | | | | | | 1. አዎ 2.አይደለም | | | | | | | | | | |  | | | | |
| 504 | | ምን አይነት ሽንት ቤት ነው የምትጠቀሙት? | | | | | | | | | 1.ሲንክ የተገጠመለት  2.ቬንትሌትድ(የአየርመናፈሻ) የተገጠመለት  3.በርብራብ የተሰራ ሽንትቤት  4.ርብራብ የሌለው(ክፍት) ሽንትቤት  5.ሌላ(ይገለፅ)------ | | | | | | | | | | |  | | | | |
| 505 | | እዚህ ከተጠቀሱት ውስጥ የትኛው በናንተ ቤት ውስጥ አለ ?  (ከአንድበላይመመለስይቻላል) | | | | | | | | | 1.ኤሌክትሪክ  4.ቴሌቪዥን  2.ሰዓት  3.ሬዲዮ  5.የሞባይል ስልክ  6.የቤትስልክ  7.ፍሪጅ  8.ጠረፔዛ  9.ወንበር  10.ሶፋ  11.አልጋ  12.ብፌ  13.መኪና  14.ሞተር ሳይክል  15.ጋሪ  16.ብስክሌት  17.ሌላ(ይገለፅ)__ | | | | | | | | | | |  | | | | |
| 506 | | ምግብ ለማብሰያ የምትጠቀሙት ምንድ ነው? (አንዱንብቻያክብቡ) | | | | | | | | | 1. ኤሌክትሪክ  2. ነጭጋዝ  3. ከሰል  4.እንጨት  5. ኩበት  6.ሌላ(ይገለፅ)_____ | | | | | | | | | | |  | | | | |
| 507 | | የምትኖሩበት ቤት ይዞታው የማን ነው? | | | | | | | | | 1.የግል/የራሳችሁ  2.የክራይ  3.ሌላ/ይጠቀስ__ | | | | | | | | | | |  | | | | |
| 508 | | በቤት ውስጥ ለምኝታ የሚያገለግል ክፍል ብዛት? | | | | | | | | | ------------በቁጥር | | | | | | | | | | |  | | | | |
| 509 | | ራሱን የቻለ የምግብ የማብሰያ ክፍል አላችሁ? | | | | | | | | | 1.በተለየ ማብሰያ ክፍል (በቤትውስጥ)  2.መኖሪያቤት ውስጥ በየትኛውም ቦታ  3.በተለየ ማብሰያ ቤት  4.ከቤትውጭ | | | | | | | | | | |  | | | | |
| 510 | | የቤቱ ወለል ከምንድ ነው የተሰራው  (እባክዎንይመልከቱት ) ? አንዱንብቻያክብቡ | | | | | | | | | 1.አፈር/ጠጠር  2.በእበት የተለቀለቀ  3.ሲሚንቶ  4.ቅርቅሃ  5.ዝጋጃ ምንጣፍ  6.ሌላካለይጠቀስ___ | | | | | | | | | | |  | | | | |
| 511 | | የቤቱ ጣራ ከምንድ ነው የተሰራው (እባክዎን ይመልከቱት ) ?  አንዱንብቻያክብቡ | | | | | | | | | 1.ከቆርቆሮ  2.ከሲሚንቶ  3.ከእንጨት  4›ከቀርቀሃ  5)ሌላ (ይገለፅ)________ | | | | | | | | | | |  | | | | |
| 512 | | የቤቱ የዉጭ ግድግዳ ከምንድ ነው የተሰራው (እባክዎንይመልከቱት ) ? አንዱንብቻያክብቡ | | | | | | | | | 1.ከድንጋይ በጭቃ  2.ከእንጨት በጭቃ  3.ከድንጋይ በሲሚንቶ  4.ሌላይጠቀስ-------------- | | | | | | | | | | |  | | | | |
| 513 | | የቤታችሁ መስኮት ከምንድ ነው የተሰራው? | | | | | | | | | 1.ከጣውላ የተሰራ መዝግያ  2.መስተዋት  3.መጋረጃ አይነት  5.ሌላ(ይገለፅ)---- | | | | | | | | | | |  | | | | |
| 514 | | ዋናው የቤተሰባችሁ የገቢ ምንጭ ምንድ ነው?  አንድመልስብቻያክብቡ | | | | | | | | | 1.እርሻ  2. እንስሳት እርባታ  3.የቅጥር ደመወዝ  4.ጥቃቅን ንግድ  5. የቀንስራ  6.ሌላ(ይገለፅ)______ | | | | | | | | | | |  | | | | |
| 515 | | የእርሻ መሬት አላችሁ? | | | | | | | | | 1. አዎ 2.የለንም | | | | | | | | | | | መልስዎ2ከሆነወደ519 ይለፉ | | | | |
| 516 | | የእርሻ መሬቱ ባለቤትነቱ የማንነ ው? | | | | | | | | | 1. የእራሳችን፣ሄክታር--------   2.የክራይ፣ሄክታር----- | | | | | | | | | | |  | | | | |
| 517 | | በአማካይ ምን ያህል ሄከታር መሬት አላችሁ? | | | | | | | | | ----------------ሄክታር | | | | | | | | | | |  | | | | |
| 518 | | በአመት በአማካኝ የምታገኙት የሰብል ምርት ስንት ይሆናል? | | | | | | | | | --------------በኩንታል | | | | | | | | | | |  | | | | |
| 519 | | የቤት እንስሳት አላችሁ? | | | | | | | | | 1. አዎ 2. የለንም | | | | | | | | | | |  | | | | |
| 520 | | የራሳችሁ የሆኑ የቤት እንሰሳት አሉ አችሁ? | | | | | | | | | 1.አዎ 2.የለም | | | | | | | | | | | መልስዎ 2 ከሆነ ወደቁ.522 | | | | |
| 521 | | ምን ያህል የሚከተሉት የቤት እንሰሳት በቤት ውስጥ ይገኛሉ?  [ከአንድ በላይ መልስ መመለስ ይቻላል] | | | | | | | | | 1.ላም______ 2. በሬ_____  3. ፈረስ-------- 4.አህያ--------  5.በቅሎ_______ 6. በግ-----------  7.ፍየል______ 8.የንብቀፎ---------  8.ሌላ(ይገለፅ)_____ | | | | | | | | | | |  | | | | |
| 522 | | ከቤተሰባችሁ መካከል የባንክ/ የቁጠባ ደብተር ያለው አለ? | | | | | | | | | 1. አዎ 2.የለም | | | | | | | | | | |  | | | | |

ጨርሻለሁ አመሰግናለሁ!
