# Supplementary material for: Short birth interval and associated factors among women who gave birth in the last three years in Dembecha district, Northwest Ethiopia
Source: PLoS One. 2022 Aug 23;17(8):e0272612. doi: 10.1371/journal.pone.0272612 (PMC9398008; doi:10.1371/journal.pone.0272612)
Supplement: S1 Appendix — (DOCX) [file pone.0272612.s003.docx]

***Appendix****:*

**Summary of wealth index measurement**

The wealth index was measured by a simplified and updated Ethiopian wealth index tool. The tool contains simplified household assets questions. Accordingly, the wealth index of the household was ranked into four quartiles (1-4). Factors with an eigenvalue greater than one were considered in the consequent analysis to characterize the variables using the varimax rotation method. For the final stage, it was observed that all items were correlated at ≥0.3 with at least one other item, but at <0.9 suggesting reasonable no Multicollinearity. Secondly, the Kaiser-Meyer-Olkin measure of sampling adequacy was 0.87, and Bartlett’s test of sphericity was significant (p <0.001). The anti-image correlation matrix and communalities were>0.5 producing four components explaining the total variance of 75.6% and overall inter-item consistency checked and it generated Cronbach alpha of 0.815. The final wealth score was ranked into four. So, the percentages of each quartile are the result of the final wealth score ranked into four quartiles.
